# Correction: Facial blushing and feather fluffing are indicators of emotions in domestic fowl (*Gallus gallus domesticus*)

**DOI:** 10.1371/journal.pone.0327223

**Published:** 2025-07-01

**Authors:** The PLOS One Editors

The *PLOS One* Editors issue this correction notice to update the ethics statement of this article [1].

Following the publication of this article [1], PLOS investigated concerns pertaining to a perceived inconsistency between the ethics approval statement reported in the article stating that ethics approval is not required for a behavioral observation study, and the methodology section reporting a capture test that involved researchers physically interacting with the chickens, which could be considered experimental.

PLOS followed up with the authors, who provided a copy of the ethics approval document #CE19–2022–1310–1, the study protocol, and letters from both the Ethics Committee for Animal Experimentation of Val de Loire and the Animals used for Scientific Purpose Unit at the French Ministry of Higher Education and Research indicating that ethics approval was not required for the manual capture of chickens. During assessment of these documents, PLOS noted that the ethics approval document #CE19–2022–1310–1 and the study protocol were dated after the study reported in [1] was conducted. The authors confirmed that these documents relate to a follow-up study that included and expanded upon the methodology reported in [1]. PLOS received confirmation from the Ethics Committee for Animal Experimentation of Val de Loire (CEEA Vdl, CEEA—019) that ethical approval was not required for the study reported in [1] as the experiments were below the minimum threshold needed for a project to require formal ethical approval.

In light of the information received by PLOS, the ethics statement of this article [1] is updated to:

The animal handling reported in this study fell below the minimum threshold required for ethical consideration by the French and European animal experimentation regulations. Therefore, the Ethics Committee for Animal Experimentation of Val de Loire (CEEA Vdl, CEEA—019) considered that ethical approval was not required for this study.
