# Supplementary material for: Facial blushing and feather fluffing are indicators of emotions in domestic fowl (Gallus gallus domesticus)
Source: PLoS One. 2024 Jul 24;19(7):e0306601. doi: 10.1371/journal.pone.0306601 (PMC11268617; doi:10.1371/journal.pone.0306601)

S1 Table. Number of images per situation and hen for M-hens and P-hens.

Table 1. Number of images per situation and hens for M-hens


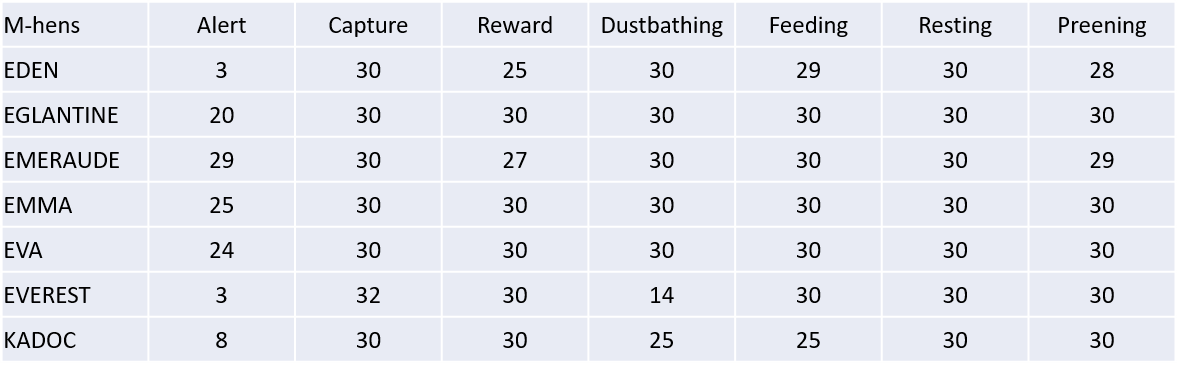


Table 2. Number of images per situation and hens for P-hens


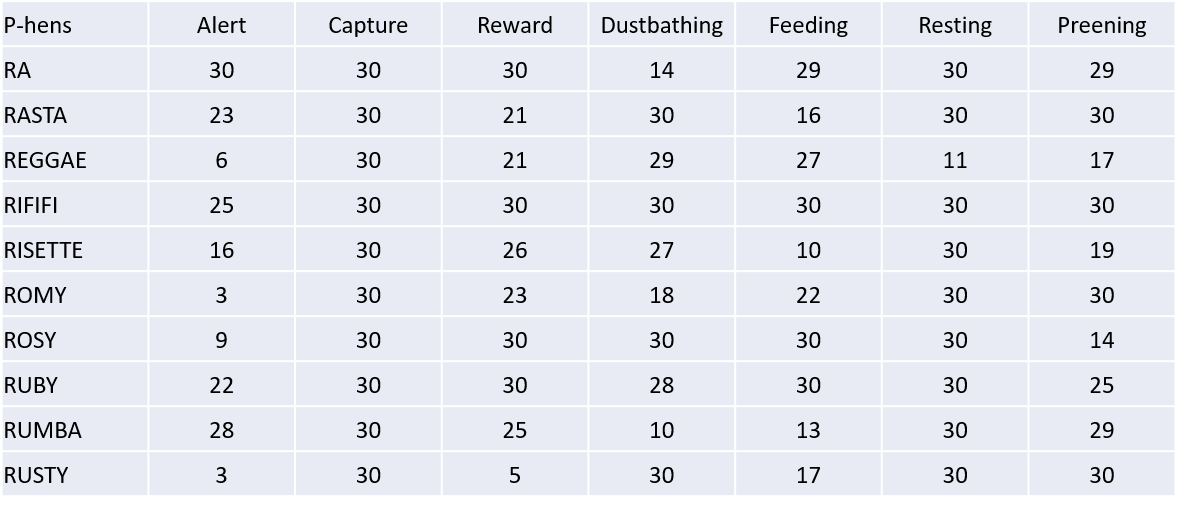

Supplement: S1 Table — (DOCX) [file pone.0306601.s001.docx]
