# Supplementary material for: Facial blushing and feather fluffing are indicators of emotions in domestic fowl (Gallus gallus domesticus)
Source: PLoS One. 2024 Jul 24;19(7):e0306601. doi: 10.1371/journal.pone.0306601 (PMC11268617; doi:10.1371/journal.pone.0306601)

S2 Fig. Redness of white feathers per situation.

Box plot (median, interquartile range and individual values) of the redness of white feathers (white) across the situations. (a) M-hens. (b) P-hens. The horizontal line represents the white theoretical value (0,33). The figures include the redness of the skin for the four ROIs (wattles, comb, cheek and ear lobes) depending on the situations (Alert, Capture, Reward, Dustbathing, Feeding, Resting, Preening), already presented in the figure 3 of the manuscript, with an additional Close situation on the right hand of the figures. For Close situation, we filmed hens resting spontaneously in close contact with the observers (distance < 50 cm, some hens being in physical contact with them or perched on their legs). This situation illustrate that bird were very confident with the observers since the skin redness was comparable to the values obtained in Resting situation.


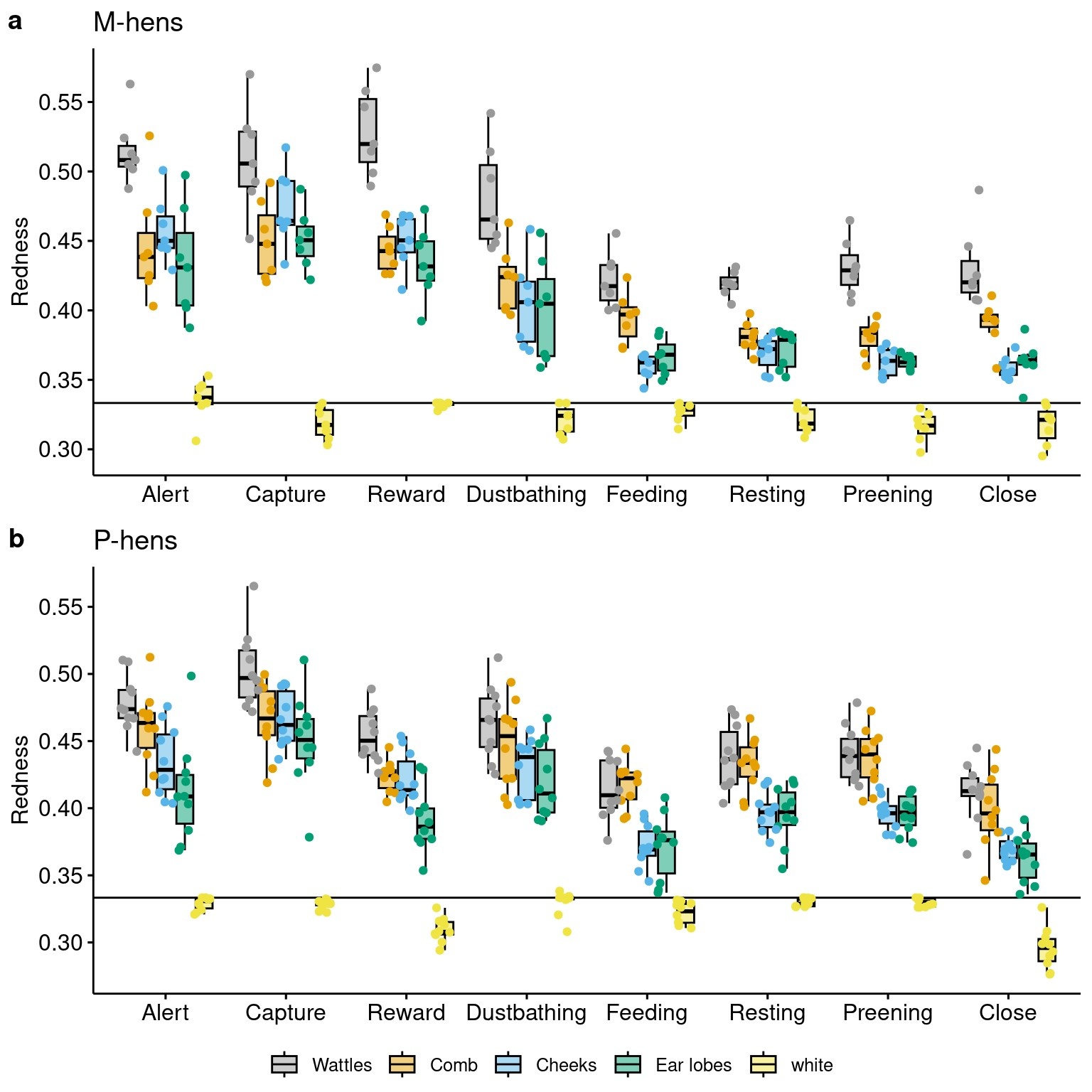

Supplement: S1 Fig — (DOCX) [file pone.0306601.s002.docx]
