# Supplementary material for: Facial blushing and feather fluffing are indicators of emotions in domestic fowl (Gallus gallus domesticus)
Source: PLoS One. 2024 Jul 24;19(7):e0306601. doi: 10.1371/journal.pone.0306601 (PMC11268617; doi:10.1371/journal.pone.0306601)
Supplement: Attachment — Submitted filename: Response_to_Reviewers.docx [file pone.0306601.s004.docx]

Dear Editor,

Please find below our responses to each point raised by the reviewers. We would like to thank the reviewers for their positive comments on our work. We think that their comments helped us to improve our manuscript.

The line numbers below refer to “Revised_Manuscript_with_Track_Changes”

**Reviewer #1:** *The authors addressed the assessment of emotions in different contexts (valence positive or negative /arousal low or high) in domestic fowl by using new behavioural indicators in domestic birds: facial blushing and feather fluffing.
The study is creative and shows interesting results which might be a first step to find applications for improving to assess welfare.

The paper is well written, so apart from minor comments detailed below.

Comments in details :
No key words?*

Key words were not asked for the submission. Key words proposed if required are: Facial expression, welfare, sentience, emotion, communication, *Gallus gallus domesticus*

*ABSTRACT:
Few information about results for feather fluffing. Is it possible to add little bit more?*

We added (lines 29-31): “Feather position also varied with the situations. Feather fluffling was mostly observed in positively valenced situations, except when hens were eating.”

*INTRODUCTION
L47: Replace our with the.*

Done line 49

*L61: Could you modify the sentence? The question is more that ethical question, is a welfare question.*

Yes, it is true. We have replaced the sentence with: “This is critical when looking at bird welfare, particularly in case of domestic species. It is also an ethical question.” (lines 63-65)

*MATERIAL and METHODS

You studied 2 strains : Pekin and Meusienne with respectively 10 and 8 hens. Is it a sample of hens within each flock? if it is the case need to be mentioned and add information how you selected the hens.*

It was not a sample of hens. No more hens were available. They represent the total number of hens that we were able to obtain for each strain at the sites where we worked. For the Meusienne group, we had planned to work with a larger number of hens, but the eggs were collected in a remote location and, unfortunately, problems arose during egg transport due to the COVID pandemic situation at that time.

To clarify that point, we added in the “Animals and sites” section: “These hens hatched on the farm and were reared in groups of mixed sexes and breeds before the experiments. Only ten female Pekin bantam from the same age and only 8 females Meusienne were available for the experiment. For both breeds, the experimental group was constituted one week before the beginning of the experiment.” (lines 125-128)

*L121 “ ..known for their calmness and closeness with humans.” add bibliographic reference.*

Two references have been added line 129 and lines 692-695.

- Husson H. Guide des races de poules. 130 races françaises et étrangères. Paris : Editions Ulmer;. 2019. pp. 232-233.

- Nuttall P. Le guide Larousse des poules et du poulailler. Bien les choisir, les nourrir et les garder en bonne santé. Edition Larousse; 2020. p. 218.

*I am not sure to understand how you proceed to organize your sequence for filming.
Did you decide to follow one specific animal? Did you wait for the specific routine behaviours?
Need more information about your method.*

We wait for specific routine behaviours. The objective was to limit as much as we could our movements to minimise bird disturbance.

We have added some precisions about our method in the “Filming the hens” section (lines 165-168): “The observers waited for the specific routine behaviours to be expressed rather than filming a single animal for a given time. This method had the advantage of minimising the movements of the observers and therefore disturbances of the bird. It also allowed filming only when the hen’s head was clearly visible.”

*L162: “hens spontaneously entered the arena”. No training or motivation stimulus to lead the animal to go in the arena? you wait all the day each animal entered in the arena? What happen with a hen which doesn’t to go inside.
Add information about that.*

Animals were very familiar with the observers. We added information how we proceed to obtain a good relationship with the hens (lines 148-150).

As mentioned in our previous manuscript (lines 184-186 in the revised manuscript), hens had been trained: “All hens had free access to the arena from approximately 36 hours before being tested. They had also been previously familiarized to eat mealworms from the dish inside the arena.”

In both groups, birds were waiting at the entrance of the arena. One of the difficulties was to be sure that only one hen entered. Those that had been already tested tried to enter again. For very few birds, one observer had to encourage them to the entrance by pushing their bottom gently when the other observer attracted the other hens. Tests were performed in 70 min then 50 min in P-hens and 50 min then 45 min in M-hens.

Lines 187-189 we added: “All birds were waiting at the entrance of the arena. It took less than 70 minutes to test all P-hens and less than 50 minutes to test all M-hens.”

*L150: hens run fast, they didn’t try to escape when you tried to catch them ? How long did you wait before to catch the next one? each capture can have an impact on the all flock.*

The first hens captured did not try to escape, but the following tried. We added lines 173: “When necessary, the hen was gently led to a corner of the field to be captured.”

It is true that each capture has an impact on the flock. We did not wait before to catch the next one to test all the birds on a short period. The delay between each capture varied between 1 and 4 minutes for P-hens and 2 and 5 minutes for M-hens, depending on the delay necessary to capture the next hen.

We added these precisions lines 177-179: “After one hen had been filmed, another one was captured. The delay to capture the next hen varied from 1 to 4 minutes in P-hens (mean = 2 min 28 s) and 2 to 5 minutes (mean = 3 min 30 s) in M-hens”.

*L174 No rings for P-hens?*

It was not necessary. Their identification was easy with their phenotypic characters. We added why we used ring for M-hens lines 203: “because their plumage colorations were very similar.”

*Image extraction, selection and analysis:
For some animals and situations, you got few images. What could be the impact on your results?*

The impact is likely low. We worked on 165 to 300 (P-hens) or 112 to 212 (M-hens) images per situation, with a mean number per hen higher than 20 images per situation, except for alert where the mean number of images was 16.5 (P-hens) and 16.0 (M-hens). Using a random sampling on our images, we observed that the use of only 20 images per bird and situation to assess skin redness was sufficient to obtain similar results*. In the Alert situation were the number of images obtained was the lowest, the number of images per hen is higher than 20 in most of the cases (P-hens: 6/10 for redness and 6/8 for feather position, and M-hens: 4/7).

* It is why in Soulet et al, 2024 (*Applied Animal Behaviour Science*, ttps://doi.org/10.1016/j.applanim.2024.106268) we used only 20 images per situation for each hen. Results obtained on 6 Sussex hens have confirmed our present results.

We have added some precisions in the manuscript lines 240-244: “We used 1710 images for P-hens (165 to 300 images per situation) and 1336 images for M-hens (112 to 210 images per situation). For some behaviours and hens the number… (S1 Table), however similar results were obtained when analyses were performed on a random sampling of 15 to 30 images per hen and situation”.

The figures below illustrate the percentage of cases (out of 1000 simulations carried out on our dataset – random sampling) where a significant difference was obtained (Wilcoxon test) between the skin redness values with n images (1<n<29) per hen and the 30 images per hen. Statistical analyses were applied on the median redness for each hen as in our study (see Statistical analysis section).

*L198: “Images came, as far as possible, from different days and behavioural sequences”. What does it mean more information.*

We clarified that point in lines 230-234.

It seems that this part on the selection of the images was unclear since Reviewer #2 also had questions on this point (LINE 199). The paragraph has been modified lines 227-237 to answer the comments of both reviewers. We hope the information added clarify the method we used. Please let us know if it is not clear enough.

*At which time the image is selected for each behaviour ? at the beginning, in the middle , at the end ?
For example dustbathing last quite a long time and I guess the redness progessed over the time.*

The selection was only based on three criteria that are mentioned lines 223-226 (193-196 in the previous manuscript): skin of comb, wattle, ear lobe and cheek visible on the image, uniform light and no direct sun on these skin areas. We added to clarify: “Selection was only based on these three criteria.” (lines 226-227). Even if some behaviours, such as dustbathing, last quite a long time, our objective was only to compare situations. It is difficult to have such a precision when selecting images (during dustbathing, birds are moving a lot, turn around, heads are often only partly visible, and as birds performed this behavior in group head is often hidden by a conspecific).

As mentioned in the Discussion (lines 488-493 in the previous manuscript), dustbathing is characterized by different phases. Redness do not progress linearly but “We observed alternation of phases of high and low redness (as well different level of feather erection), likely due to an alternation of high and low arousal.” (lines 492-494 in the revised manuscript).

*L271 : put ad libitum in italic*

Done (line 311)

*RESULTS
L291-292: The chi² analyze is not mentioned in statistical section. Please mention it.*

As mentioned in statistical section lines 274-275, we used “a binomial Generalized Linear Mixed Model (GLMM) (lme4 package [40])”. In binomial GLMs, variables are tested for significance using the chi2 distribution (instead of the Fisher distribution in the Gaussian case), hence the Chi² statistic presented in the text. However, no Chi² test on a contingency table was performed.

*L324 -337: Please write a sentence for the 3 others ROI in different situations.*

Done lines 376-377. We added “For the three other ROI, results were similar but situations were more difficult to discriminate statistically, especially for wattles and comb.”

*REFERENCES
L715: please correct this reference.*

Done (lines 797-800)

***Reviewer #2****: This is a very interesting article investigating the redness of skin as a measure of valence and arousal in chickens. My edits and comments are detailed by line number below.*

*Abstract – Please add at least one statement about the results related to feather position and whether that was or was not repeatably indicative of affective state and why.*

Done lines 29-31: “Feather position also varied with the situations. Feather fluffing was mostly observed in positively valanced situations, except when hens were eating.”

*LINE 22: Please revise “domestic hens” to “female chickens” here and throughout. “Hen” can refer to females from many different domesticated poultry species.*

*LINE 84: See LINE 22.*

Revised throughout the manuscript. Domestic hens have been replaced by “female chickens” (lines 116, 121, 427, 501, 508), “female domestic fowl” (lines 467, 473) or “domestic fowl” (lines 22, 238) depending on the sentences.

*LINE 88: Please revise “rustic hens” to “hens from two rustic chicken breeds”.*

Done line 92.

*LINE 128: What were the outdoor temperatures during video recording? Temperature can affect skin color.*

This is an interesting point. We added lines 193-194 “The outdoor temperatures were approximately 25°C (P-hens) and 27°C (M-hens) during the video recordings, and the weather was dry and mainly sunny.” and we have discussed this point more thoroughly than before in the Discussion section. See also answer to Reviewer #3, L481-482, for references and to complete the answer.

We found no references showing that temperature can affect skin colour (or can induce rapid changes in skin colour). The only reference we found concerned the effect of the temperature on skin temperature measured by infrared thermography. However, it seems there is no direct link between redness and skin temperature. In the situations where skin redness was high in our study (capture, reward), skin temperature decreases in the literature (when hens are tested in similar situations). See answer to Reviewer #3, L481-482.

We did not register ambient temperature precisely (hour by hour throughout a day) as we noticed no effect of the temperature on skin redness of our birds. As mentioned lines 533-534 and shown on the video (supplementary material S3), the phenomenon of facial skin flushing is transient. The colour of the face can redden or lighten in a few seconds or minutes depending on the duration of the stimulation. As ambient temperature does not vary within these short laps of time it cannot explain the differences observed. To illustrate this point, you can find below pictures of the same bird at rest (left) and during the capture test (right) taken approximately 15 minutes apart. The sentence is now: “… the blushing measured in our study appears in few seconds and is transitory. It lasts a few seconds or minutes depending on the duration of the stimulus inducing the reaction (a video can be seen on S3 Video), contrary to temperature variations during a day.” (lines 533-536).

To consider the potential effects induced by working on birds living outside in semi-natural conditions (including weather variations), we took several precautions. It seems that they did not appear clearly enough in our manuscript.

1- Temperature variations were limited when films were performed. Information added line 192: “Films were performed between 10:00 and 18:00 to avoid sunrise and sunset (at this season the sun is high in the sky and temperature variations are limited;...”

2- All the situations were filmed both in the morning and the afternoon and across the successive days of the observational period (3 weeks). “Each routine behaviour (situations) was filmed both in the morning and the afternoon and across the successive days of the observational period” added lines 168-169.

- We worked on 30 images for each hen and situation which means 100 to 300 images per situations. Precisions on the methods added lines 227-237 and 240-244.

*LINE 173: Please remove “…” after “size”.*

Done line 202.

*LINES 175-189: This paragraph should be moved to the discussion section.*

We think it is necessary to explain why these situations were selected for this study (see for example the comment Reviewer #3 L437-447). As this change was not required by the two other reviewers, we preferred not to move this paragraph to the discussion section.

*LINE 197: Please identify/describe the version of Python and script used to select the 30 random images.*

The script is freely available on https://forgemia.inra.fr/projetred/red_project (sampling_files.py”). This information had been added line 229. We used Python version 3.8

*LINE 199: Please explain how sampling was multiplied to minimize bias. How may times?*

It referred to the 30 images for each hen and situation. As this part on images selection appeared unclear (see also Reviewer #1 L198 comment), it has been rewritten to clarify (lines 226-237).

*LINE 415: What was the high percentage?*

Values of the medians (provided Table 1) have been added lines 455-456: “… (median at 40 % or more in M-hens and 56 % or more in P-hens).”

*LINES 418-419: Ruffled head feathers can also indicate sickness behavior. Be cautious with this interpretation.*

Yes, it is true. We have modified our sentence and included this information lines 459-462: “Fluffing of head feathers appears to be a relevant indicator of positive emotional states in situation where birds are healthy. Indeed, feather fluffing could also signal sickness in birds (in this case, birds are often prostrated and feathers are in bad conditions).”

*LINES 428 and 429: Usually “mutual preening” is referred to as “allopreening”.*

Changed lines 471 and 472.

*LINE 481: Ambient temperature needs to be reported in the materials and methods section, especially if this statement is going to be made.*

Done lines 193-194. See answer to the previous comment *LINE 128*.

We also added some methodological considerations and discussed more deeply why the temperature variations cannot explain our results: “The naturalistic approach also makes difficult to control with precision for temperature, humidity or wind; it is why we worked on a lot of images (30 images spread along the observational period). This allows to consider the variability that could have been induced by the environmental conditions. However, temperature did not vary very much during our study and our results cannot be explained by thermoregulation variations. Indeed, the blushing measured in our study appears in few seconds and is transitory. It lasts few seconds or minutes depending on the duration of the stimulus inducing the reaction (a video can be seen on S3 Video), contrary to temperature variations during a day.” (lines 529-536).

In an outdoor environment, the hens are free to move from a sunny to a shady spot (under a tree) or to protect themselves from the wind. Consequently, it is not possible to control these parameters for each of the films made.

See also answer to Reviewer #3 L481-482.

*LINE 515 and 516: Please combine these two sentences.*

Done line 578.

***Reviewer #3****: Overall comment:
The proposed manuscript is related to understanding emotionality in chickens using changes in facial expressions. This study, if replicated/repeated, could add valuable information in assessment of affective state as an indicator of bird welfare. However, the biggest limitation of this study is the fact that it is conducted in small number of birds in variable environmental conditions. The authors have added limitations of the study in the discussion section of the manuscript but without the knowledge of the ambient temperature and humidity conditions at two barns used in the study (at the very least), interpretation of the data cannot be complete.*

We agree that we worked on small number of birds and in variable environmental conditions. But results obtained are replicated on two breeds: Pekin (n=10) and Meusienne (n=8). These results have also been replicated in a third breed: Sussex (n=6) and recently published in *Appl Anim Behav Sci* (https://doi.org/10.1016/j.applanim.2024.106268). A reference to this study has been added line 517 (Reference added lines 776-779): “However, we have already replicated the data on the two breeds studied in this experiment (Pekin, Meusienne) in another breed (Sussex, [59]), …”

We also obtained similar results on another group of adult Pekins (n=12); a study that will be submitted shortly.

See below for environmental conditions

*Specific comments:
Material and methods:*

*- L119-120: Provide evidence to the prosocial claims of the hens used in the study.*

Done line 129

*- Provide temperature/humidity conditions between both farms.*

Temperature/humidity conditions have been added lines 193-194 (see answer to Reviewer #2 LINE128). During the experiment, birds were outside, it is therefore not possible to provide precise measures. We were not in barns during the observations as written in “Overall comments”. Birds had permanent access to a small henhouse and were never inside except at night. To clarify this point, dimensions of the henhouse had been added lines 135-139 to clarify this point “… (P-hens: 1.20 m width, 0.85 m length x 0.70 m height, with access to an additional space 0.35 m x 0.85 m x 0.45 m, henhouse 10 cm above the ground; M-hens: 1.54 m width, 0.80 m length, 0.70 m height in front, 0.40 m height in back, with a platform at the entrance: 0.80 m length, henhouse 35 cm above the ground) …”

*- L137: Elaborate what ‘activation of white balance function’ does to the results of the study.*

The sentence has been modified to explain why we use this function: “… automatic white balance function activated to limit variations that could occur between sunny and more cloudy periods” (lines 152-153).

*- L176: provide context behind ‘alert’ behavior observed in the methods section as well. Was ‘alert’ behavior displayed in response to the actual presence of the Common buzzard?*

This information has been added in the Material and methods section (lines 160-162): “… Alert (immobile posture with raised neck following a sudden event: common buzzard in the sky, emitting vocalizations in most cases; dog barking; tractor noise; plane noise passing by at low altitude; alert vocalizations of other fowl in the vicinity).

*- L178: consider changing ‘affective state’ to ‘emotional state’*

We have changed “generate affective states associated with fear” with “generate fear-related emotions” (line 207).

Discussion:

- L437-447: We have to be careful about the assumption that dustbathing behavior is rewarding to hens in absence of neurophysiological data from hens performing dustbathing. Could the discussion also explore the possibility that dustbathing yielded intermediate results because the behavior elicits neutral valence rather than the presumed positive valence?

Even if neurobiological data are lacking for dustbathing, there are several behavioural data that support the assumption that dustbathing behaviour is rewarding. According to Mendl et al (2010), positive emotions are associated with appetitive motivational states (anticipation) and reward-seeking behaviours. Dustbathing behaviour fulfil these two conditions (McGrath et al 2016). This point is indicated lines 211-212 of the Material and methods section: “Domestic fowl work for access to mealworms or to dusty substrate to perform dustbathing, and show positive anticipation behaviours [30-32])”. According to Widowski and Duncan, 2000, the motivation to dustbathe is driven not only by the need to clean the plumage but also by the possible pleasure (positive affective state) engendered by performing this behaviour. We modified the previous sentence: “… but also neurobiological substrates in case of mealworms.” (lines 210-211)

The possibility that dustbathing yielded intermediate results because the behaviour elicits neutral valence rather than the presumed positive valence has been added in the discussion lines 487-489: “In case of dustbathing, it seems unlikely that this behaviour had a neutral valence. Birds are highly motivated to dustbathe and according to some authors dustbathing behaviour probably elicits a feeling of pleasure [32,52]. That said, it is also possible that these intermediate redness values are the result of the dynamics of arousal level ….”

32. McGrath N, Burman O, Dwyer C, Phillips CJ. Does the anticipatory behaviour of chickens communicate reward quality? Appl. Anim. Behav. Sci. 2016; 184: 80–90. https://doi.org/10.1016/j.applanim.2016.08.010

52. Widowski, T. M., & Duncan, I. J. (2000). Working for a dustbath: are hens increasing pleasure rather than reducing suffering? Applied Animal Behaviour Science, 68(1), 39-53.

- L481-482: This discussion should include at the very best ambient temperature and humidity data from both farms (M and P house). Also, there are studies to suggest changes in facial surface temperatures under different thermal conditions (For example - Kim, N. Y., Kim, S. J., Oh, M., Jang, S. Y., and Moon, S. H. 2021. Changes in facial surface temperature of laying hens under different thermal conditions. Anim. Biosci. 34(7): 1235-1242.). As this study was conducted in barn-type environment with less control over temperature and humidity, the effects of these factors on the results cannot be negated.

We provided more information on our method (lines 226-237 & 240-244) that we hope will convince the reviewer that these conditions do not interfere with our results. See answer to Reviewer #2 LINE 128 and Reviewer #1 L198, and please see the video provided as supplementary data – S3 Video).

We have completed our discussion on the potential influence of ambient temperature on our results as suggested. See lines 529-545. “

- We added how we proceed to consider the variability induced by the fact that birds were in semi-natural conditions: “The naturalistic approach also makes difficult to control with precision for temperature, humidity or wind; it is why we worked on a lot of images (30 images spread along the observational period). This allows to consider the variability that could have been induced by the environmental conditions.”

- Furthermore, we discussed differences that probably exist between measures of skin redness and skin temperature by infrared thermography: “… the blushing measured in our study appears in few seconds and is transitory. It lasts a few seconds or minutes depending on the duration of the stimulus inducing the reaction (a video can be seen on S3 Video), contrary to temperature variations during a day. According to Ioannou et al (2014; [60]), “thermal signal development as a result of vascular change, or muscular activity”, measured using thermal infrared imaging, “is rather sluggish compared with other measures of physiological arousal”. Facial surface temperature measured by infrared thermography varied with contrasted thermal environments (10, 20 and 30 °C; [61]). However, such variations of the temperature did not exist during our observations. Furthermore, it seems that the skin redness we measured and skin temperature measured by infrared thermography do not measure the same phenomenon. Indeed, while we obtained high redness in Reward and Capture situations, the comb temperature dropped in laying hens eating mealworms [62] or in response to handling [63].”

We added 4 references: (lines 780-792)

60 Ioannou S, Gallese V, Merla A. Thermal infrared imaging in psychophysiology: potentialities and limits. Psychophysiol. 2014; 51: 951-963. https://doi.org/10.1111/psyp.12243

61 Kim NY, Kim SJ, Oh M, Jang SY, Moon SH. Changes in facial surface temperature of laying hens under different thermal conditions. Anim. Biosci. 2021; 34: 1235-1242. https://doi.org/10.5713/ab.20.0647

62 Moe RO, Stubsjøen SM, Bohlin J, Flø A, Bakken M. Peripheral temperature drop in response to anticipation and consumption of a signaled palatable reward in laying hens (Gallus domesticus). Physiol. Behav. 2012; 106: 527-533. https://doi.org/10.1016/j.physbeh.2012.03.032

63 Edgar JL., Nicol CJ., Pugh CA, Paul ES. Surface temperature changes in response to handling in domestic chickens. Physiol. Behav. 2013; 119: 195-200. https://doi.org/10.1016/j.physbeh.2013.06.020
